# *In vivo* kinetics of *Wolbachia* depletion by ABBV-4083 in *L*. *sigmodontis* adult worms and microfilariae

**DOI:** 10.1371/journal.pntd.0007636

**Published:** 2019-08-05

**Authors:** Marc P. Hübner, Marianne Koschel, Dominique Struever, Venelin Nikolov, Stefan J. Frohberger, Alexandra Ehrens, Martina Fendler, Iliana Johannes, Thomas W. von Geldern, Kennan Marsh, Joseph D. Turner, Mark J. Taylor, Stephen A. Ward, Kenneth Pfarr, Dale J. Kempf, Achim Hoerauf

**Affiliations:** 1 Institute for Medical Microbiology, Immunology and Parasitology, University Hospital Bonn, Germany; 2 Global Pharmaceutical Research and Development, AbbVie, North Chicago, Illinois, United States of America; 3 Franciscan Institute for World Health, Franciscan University, Steubenville, Ohio, United States of America; 4 Centre for Drugs and Diagnostics, Department of Tropical Disease Biology, Liverpool School of Tropical Medicine, Liverpool, United Kingdom; 5 German Center for Infection Research (DZIF), partner site Bonn-Cologne, Bonn, Germany; National Institutes of Allergy and Infectious Diseases, NIH, UNITED STATES

## Abstract

Depletion of *Wolbachia* endosymbionts of human pathogenic filariae using 4–6 weeks of doxycycline treatment can lead to permanent sterilization and adult filarial death. We investigated the anti-*Wolbachia* drug candidate ABBV-4083 in the *Litomosoides sigmodontis* rodent model to determine *Wolbachia* depletion kinetics with different regimens. *Wolbachia* reduction occurred in mice as early as 3 days after the initiation of ABBV-4083 treatment and continued throughout a 10-day treatment period. Importantly, *Wolbachia* levels continued to decline after a 5-day-treatment from 91.5% to 99.9% during a 3-week washout period. In jirds, two weeks of ABBV-4083 treatment (100mg/kg once-per-day) caused a >99.9% *Wolbachia* depletion in female adult worms, and the kinetics of *Wolbachia* depletion were recapitulated in peripheral blood microfilariae. Similar to *Wolbachia* depletion, inhibition of embryogenesis was time-dependent in ABBV-4083-treated jirds, leading to a complete lack of late embryonic stages (stretched microfilariae) and lack of peripheral microfilariae in 5/6 ABBV-4083-treated jirds by 14 weeks after treatment. Twice daily treatment in comparison to once daily treatment with ABBV-4083 did not significantly improve *Wolbachia* depletion. Moreover, up to 4 nonconsecutive daily treatments within a 14-dose regimen did not significantly erode *Wolbachia* depletion. Within the limitations of an animal model that does not fully recapitulate human filarial disease, our studies suggest that *Wolbachia* depletion should be assessed clinically no earlier than 3–4 weeks after the end of treatment, and that *Wolbachia* depletion in microfilariae may be a viable surrogate marker for the depletion within adult worms. Furthermore, strict daily adherence to the dosing regimen with *anti-Wolbachia* candidates may not be required, provided that the full regimen is subsequently completed.

## Author summary

Onchocerciasis and lymphatic filariasis represent debilitating neglected tropical diseases that are caused by parasitic filarial nematodes. Current efforts to eliminate onchocerciasis are hampered by the lack of drugs that lead to permanent sterilization of the adult worms or provide a macrofilaricidal effect, i.e. kill the adult worms. In the past, doxycycline has been shown to deplete *Wolbachia* endosymbionts of filarial nematodes, leading to permanent sterilization and macrofilaricidal efficacy in filariae causing both onchocerciasis and lymphatic filariasis. However, contraindications and a requirement for at least 4 weeks of doxycycline treatment impair its broader use, creating a need for drugs with a shorter treatment regimen and potentially fewer contraindications. ABBV-4083 is such an anti-*Wolbachia* candidate that was efficacious in several filarial animal models and has recently been tested in a phase 1 clinical trial. The present studies addressed several points that are important for subsequent phase 2 clinical trials, namely the comparison of once vs. twice-per-day treatments, the impact of missed treatments, and a comparison of the kinetics of *Wolbachia* depletion in adult worms and microfilariae, the latter of which has the potential to be a surrogate indicator to avoid the necessity of surgically removing nodules with adult worms at repeated time points.

## Introduction

Onchocerciasis and lymphatic filariasis are neglected tropical diseases that are caused by the filarial nematodes *Onchocerca volvulus* and *Wuchereria bancrofti*, *Brugia malayi* and *Brugia timori*, respectively [1]. During onchocerciasis vision loss and severe dermatitis can occur, whereas one third of lymphatic filariasis patients develop lymphedema and hydrocele [2, 3]. Due to the severity of these debilitating diseases that present a huge socioeconomic problem in endemic countries, the World Health Organization (WHO) has targeted both diseases for elimination [2, 3]. Therefore, mass drug administrations have been performed over the last decades with ivermectin for onchocerciasis in sub-Saharan Africa, ivermectin plus albendazole for lymphatic filariasis in sub-Saharan Africa, and diethylcarbamazine (DEC) plus albendazole for lymphatic filariasis outside of Africa [2, 3]. However, these regimens are mainly microfilaricidal, leading to the loss of the filarial offspring, the microfilariae. They also temporarily inhibit the embryogenesis of the female adult worms, but do not kill the adult worms, i.e. produce a macrofilaricidal effect. Therefore, these treatments need to be given on an annual or biannual basis to reduce transmission of the filariae for the reproductive life-span of the female adult worms, which can be as long as 15 years for onchocerciasis. Recently, moxidectin, which demonstrates a prolonged clearance of microfilariae in comparison to ivermectin, was registered as a new drug for onchocerciasis, providing the potential to more effectively reduce transmission between treatment rounds and accelerate elimination of onchocerciasis [4]. Furthermore, administration of a triple therapy consisting of ivermectin, DEC and albendazole was introduced for lymphatic filariasis, and it is now recommended by the WHO in areas non-endemic for onchocerciasis [5], as it leads to a prolonged depletion of the microfilariae and may also provide macrofilaricidal efficacy [6, 7]. However, potential drug-induced serious adverse events in areas co-endemic for the filarial nematode *Loa loa* in the case of moxidectin as well as *L*. *loa* and *O*. *volvulus* [8] in the case of the triple therapy, may hamper the implementation of those treatments in those areas. Furthermore, with lower endemicities of onchocerciasis and lymphatic filariasis, the cost effectiveness of community-based MDA programs is reduced [9], and modelling studies suggest that the reduction of required treatment rounds by triple therapy will be less significant in areas of lower endemicity [10]. Therefore, new macrofilaricidal drugs are needed for case management and for clearance of residual foci in order to eliminate onchocerciasis and lymphatic filariasis.

Such macrofilaricidal efficacy can be achieved by drugs that target *Wolbachia* endosymbionts, which are present in most human pathogenic filariae, including those causing lymphatic filariasis and onchocerciasis, but, notably, not in *L*. *loa*. Treatment of either onchocerciasis or lymphatic filariasis patients with doxycycline for 4–6 weeks has been shown to deplete *Wolbachia* bacteria, leading to permanent sterilization of the female worms and providing a slow and therefore safe macrofilaricidal effect [11–16]. However, the need for prolonged treatment (at least 4 weeks) and contraindications in children under age 8 and in pregnant and lactating women prevent the broader use of doxycycline for filarial diseases.

ABBV-4083 is a tylosin analogue which has improved potency and oral bioavailability compared to tylosin and has been shown to be efficacious in rodent models using the filarial nematodes *Litomosoides sigmodontis*, *B*. *malayi* and *Onchocerca ochengi* [17, 18]. Thus, ABBV-4083 represents a promising anti-*Wolbachia* candidate, and its safety in humans has recently been assessed in a phase 1 clinical trial (https://www.dndi.org/diseases-projects/portfolio/abbv-4083/).

In the current studies we addressed several points that are important for informing the design of phase 2 clinical trials of anti-*Wolbachia* candidates. These involved i) the determination of the kinetics of *Wolbachia* depletion in adult worms during and after treatment; ii) the investigation of alternate dosing regimens, i.e. the comparison of a once (QD) and twice daily (BID) treatment; iii) the assessment of whether *Wolbachia* depletion in microfilariae can be used as surrogate marker for the depletion in adult worms; and iv) the impact of a later catch-up of missed treatment days. For these experiments we used the *L*. *sigmodontis* rodent model [19, 20], as *L*. *sigmodontis* harbors *Wolbachia* endosymbionts and was previously used for pre-clinical studies of direct-acting and *Wolbachia*-targeting drug candidates, with good predictivity of the parasitological outcomes of later doxycycline clinical trials [21–27].

In the *L*. *sigmodontis* model, infective L3 larvae are transmitted during the blood meal of the tropical rat mite *Ornithonyssus bacoti*. The larvae migrate to the thoracic cavity, where they molt into adult worms within 30 days after infection (dpi) [28]. Starting ~8 weeks after infection, female worms release microfilariae, which circulate in the peripheral blood. Experiments were performed in *L*. *sigmodontis*-infected mice with treatment start after the molt into adult worms (35/36dpi) and in *L*. *sigmodontis*-infected jirds after the development of microfilaremia (3–4 months after infection). Two different rodent hosts were chosen, as only 50% of BALB/c mice develop microfilaremia, which is comparable to human lymphatic filariasis [29], and BALB/c mice clear the infection around 100 dpi [20]. In contrast, the susceptibility of jirds to infections with *L*. *sigmodontis* is increased, leading to the development of microfilaremia in the majority of animals and infections that last for more than one year [20]. Thus, jirds were used in our long-term experiments investigating microfilariae and embryogenesis, whereas mice were initially used to test the drug-efficacy against the female adult worms.

Our studies reveal that *Wolbachia* reduction occurs as soon as 3 days after treatment onset and continues during the weeks following the end of treatment. *Wolbachia* depletion by ABBV-4083 was not significantly improved by BID treatment in comparison to QD treatment. The kinetics of *Wolbachia* depletion in microfilariae coincide with the *Wolbachia* depletion in female adult worms, indicating its value as potential early clinical marker of efficacy. Finally, missed treatments could be given at later time points without impairing the *Wolbachia* depletion and parasitological outcome.

## Materials and methods

### Ethics statement

All animal experiments were performed in accordance with the European Union Directive 2010/63/EU and were approved by the Landesamt für Natur, Umwelt und Verbraucherschutz, Cologne, Germany (AZ 84–02.04.2015.A507). Animal welfare was scored on a scale from A-C for symptoms considering appearance, injuries, weight loss, and behavior. A score of A required daily observations of the symptoms, a score of B, consultation of the project leader or a veterinarian, and a score of C the immediate euthanization of the affected animal. For euthanization, animals were exposed to an overdose of isoflurane.

### Test agents

Compound ABBV-4083, Lot 2263872–0, was used for testing and provided by AbbVie. Doxycycline (Sigma Aldrich, St. Louis, MO, USA) was used as control and was stored at 4°C and suspended shortly before application in distilled water. ABBV-4083 was also stored at 4°C and homogenized in 0.5% hydroxypropylmethylcellulose/0.02% Tween 80 (Sigma Aldrich) at 4°C overnight on a magnetic stirrer. This preparation was aliquoted, stored at -20°C and used for up to 5 subsequent treatment days.

### Animals

Female jirds (*Meriones unguiculatus*) and female BALB/c J mice were obtained from Janvier (Saint-Berthevin, France) and were housed in individually ventilated cages at the animal facility of the Institute of Medical Microbiology, Immunology and Parasitology, University Hospital Bonn, with free access to food and water. The animals were maintained on a 12h day/night cycle at 20–26°C and a humidity of 30–70%. For enrichment, animals were provided wooden sticks and nestlets.

### Study design of mice infected with *L*. *sigmodontis*

Female BALB/c mice (8–10 weeks old) were naturally infected by exposure to mites (*O*. *bacoti*) containing *L*. *sigmodontis* L3 larvae. The same batch of mite-containing bedding was used to infect all animals of one study design at one time point as previously described [30]. Treatment started 35 or 36dpi for all study designs in mice and all treatments were given by oral gavage at the indicated doses either QD or BID.

In total, three mouse studies were performed:

Mouse study I: Four experimental groups were included in this study. Two groups received 75mg/kg QD ABBV-4083 (n = 6/group) for 3 or 7 days, whereas two additional groups were treated QD for 3 or 7 days with an equal volume of vehicle (n = 6). In this experiment blood was obtained 1 and 7 h after the first and last morning gavage and pipetted onto DBS filter cards (Whatman 903 Protein saver card, Sigma-Aldrich, Germany) for pharmacokinetic analyses. Vehicle and ABBV-4083 groups were euthanized 38 and 42dpi.

Mouse study II: Ten experimental groups were included in this study. Two groups received 75mg/kg QD ABBV-4083 (n = 6/group) for 5 days, two groups received 75mg/kg QD ABBV-4083 (n = 6/group) for 10 days and two groups 75mg/kg BID ABBV-4083 (150mg/kg/day; n = 6/group) for 5 days. Doxycycline (n = 6) was administered at the human bioequivalent dose of 40mg/kg BID for a suboptimal duration of 10 consecutive days. Three groups of control mice were treated once daily for 5 and 10 days with an equal volume of vehicle (n = 6). Blood was obtained by puncture of the facial vein 1 and 7 h after the last morning gavage and pipetted onto DBS filter cards for pharmacokinetic analyses. Necropsy and further analyses were performed one day after treatment end at 40, 45, and 63dpi (5, 10 and 28 days after treatment start). Vehicle-treated mice were euthanized 40, 45, and 63dpi, doxycycline controls at 63dpi.

Mouse study III: Five experimental groups were included in this study. Three groups received 43, 50, or 75mg/kg QD ABBV-4083 (n = 5/group) for 5 days, whereas one group was treated for 5 days with 43mg/kg BID ABBV-4083 (n = 5/group). Controls received an equal volume of vehicle twice a day for 5 days (n = 5). In this experiment blood was obtained 2, 8 and 24 h after the first morning gavage (before the 8h and 24h treatment) and pipetted onto DBS filter cards for pharmacokinetic analyses. Vehicle and ABBV-4083 groups were euthanized 41dpi.

### Study design of jirds infected with *L*. *sigmodontis*

Female jirds (8–9 weeks old) were naturally infected with *L*. *sigmodontis* L3 larvae by exposure to infected *O*. *bacoti* mites, using the same batch of mite-containing bedding to infect all animals of one study. At 11 weeks after infection (wpi), infected jirds were checked for peripheral microfilarial counts and microfilaremic animals were allocated to the different treatment groups. Treatment of jirds was initiated 12-15wpi and all treatments were given by oral gavage.

Jird study I: Six experimental groups were tested in this study. Group 1 received 100mg/kg QD ABBV-4083 for 7 consecutive days (n = 6), and groups 2–4 received 100mg/kg QD ABBV-4083 for 14 consecutive days (n = 6 per group). Group 5 received 40mg/kg BID doxycycline for a suboptimal duration of 14 consecutive days (n = 6). Group 6 received an equal volume of ABBV-4083 vehicle and was used as control (n = 6). Necropsy of group 1 was performed 1 week after treatment start (wpt), group 2 at 2wpt, group 3 at 4wpt and groups 4–6 at 14wpt.

Jird study II: Seven experimental groups were tested in this study. Groups 1–6 received ABBV-4083 at 25mg/kg QD for 7, 10 or 14 consecutive days (n = 6–7 per group), 7 days with missed treatments on day 6 and 8 (n = 6), or 14 days with missed treatments on day 6, 8, 13 and 15 (n = 6). Missed treatments were given the following days so that the animals received 7 or 14 treatments in total over a period of 9 or 18 days, respectively. Controls received an equal amount of vehicle once per day for 14 days (n = 6) and as a positive control jirds were treated for 14 days with 50mg/kg QD ABBV-4083 (n = 6). Necropsies were performed at 8wpt.

### Assessment of adult worm and microfilariae counts

At necropsy infection of mice and jirds was confirmed by flushing the pleural cavity with 1ml PBS and by screening for adult worms in the thoracic cavity as well as the peritoneum. Isolated worms were separated, sex determined, counted and individually frozen for subsequent *Wolbachia* analysis or stored in 70% ethanol for analysis of embryogenesis.

For jird experiments, peripheral blood microfilariae levels were quantified at 1- to 2-week intervals by microscopy starting at 11wpi until the day of necropsy. For blood collection, the vena saphena was punctured and 10μL of peripheral blood were directly transferred into 190μl of red blood cell lysis buffer (BioLegend, San Diego, CA, USA) and stored at 4°C until analysis. After resuspension, 10μl of the suspension were transferred to a microscopic slide and microfilariae were counted from the complete slide using a microscope. If less than 10 microfilariae were counted, the tubes were centrifuged at 400g for 5 minutes, the supernatant was discarded, and the pellet resuspended and completely transferred to a microscope slide for counting.

### Quantification of *Wolbachia*

Depletion of endosymbiotic *Wolbachia* was determined by qPCR using primers for *Wolbachia* single copy gene *ftsZ* (GenBank Accession No.: AJ010271) and normalized to the *L*. *sigmodontis* actin gene (*act*) (GenBank Accession No.: GU971367) as previously described [27]. If present, 10 female worms per animal were frozen at -20°C for later analysis of the *Wolbachia ftsZ*/*act* ratio by duplex real-time PCR using Qiagen’s QuantiNova on a Rotorgene Q 5-Plex (Qiagen, Hilden, Germany). The PCR was performed in triplicate. The following primer pairs (MicroSynth; Switzerland) and TaqMan probes (biomers; Germany) were used: LsFtsZ FW cgatgagattatggaacatataa, LsFtsZ RV ttgcaattactggtgctgc, LsFtsZ TQP 5’6-FAM cagggatgggtggtggtactggaa 3’TAMRA, LsActin FW atccaagctgtcctgtctct, LsActin RV tgagaattgatttgagctaatg, LsActin TQP 5’HEX actaccggtattgtgctcgatt 3’TAMRA. The qPCR consisted of 45 cycles with a melting temperature of 95°C for 5 sec and an annealing temperature of 58°C for 30 sec. The standard curve used was a mix of LsFtsZ and LsActin plasmids.

For *Wolbachia* quantification from microfilariae, 50μL of peripheral blood were lysed with 950μL RBC lysis buffer (Biolegend) at room temperature for at least 5 minutes. After centrifugation at 400g, the supernatant was discarded, and the pellet resuspended in 200μl AE buffer (Qiagen). A known number of peripheral blood microfilariae (if present 10–30 microfilariae) were stored in AE-buffer at 4°C until analysis. The PCR was performed as described for the adult worms. As microfilariae are less variable in size as adult worms, *ftsZ* values per microfilaria are shown.

### Statistics

ABBV-4083 efficacy was determined based on the reduction of *Wolbachia ftsZ*/*act* from female adult worms (primary efficacy parameter). Secondary efficacy parameters included in jird experiments the reduction of *Wolbachia ftsZ* gene from microfilariae, the inhibition of embryogenesis and the clearance of microfilariae. Statistical analyses were performed using GraphPad Prism software Version 8.02 (GraphPad Software, San Diego, USA). Distribution of data was tested for normality by d‘Agostino & Pearson test. Differences between multiple groups that were not normally distributed were tested for statistical significance using Kruskal-Wallis followed by Dunn’s multiple comparison test. Normally distributed data of multiple groups were tested for statistical significance using 1-way-ANOVA followed by Sidak’s multiple comparison test. p values ≤ 0.05 were considered statistically significant.

## Results

### Kinetics of *Wolbachia* decline in *L*. *sigmodontis* during and after ABBV-4083 treatment

**Fig 1:**
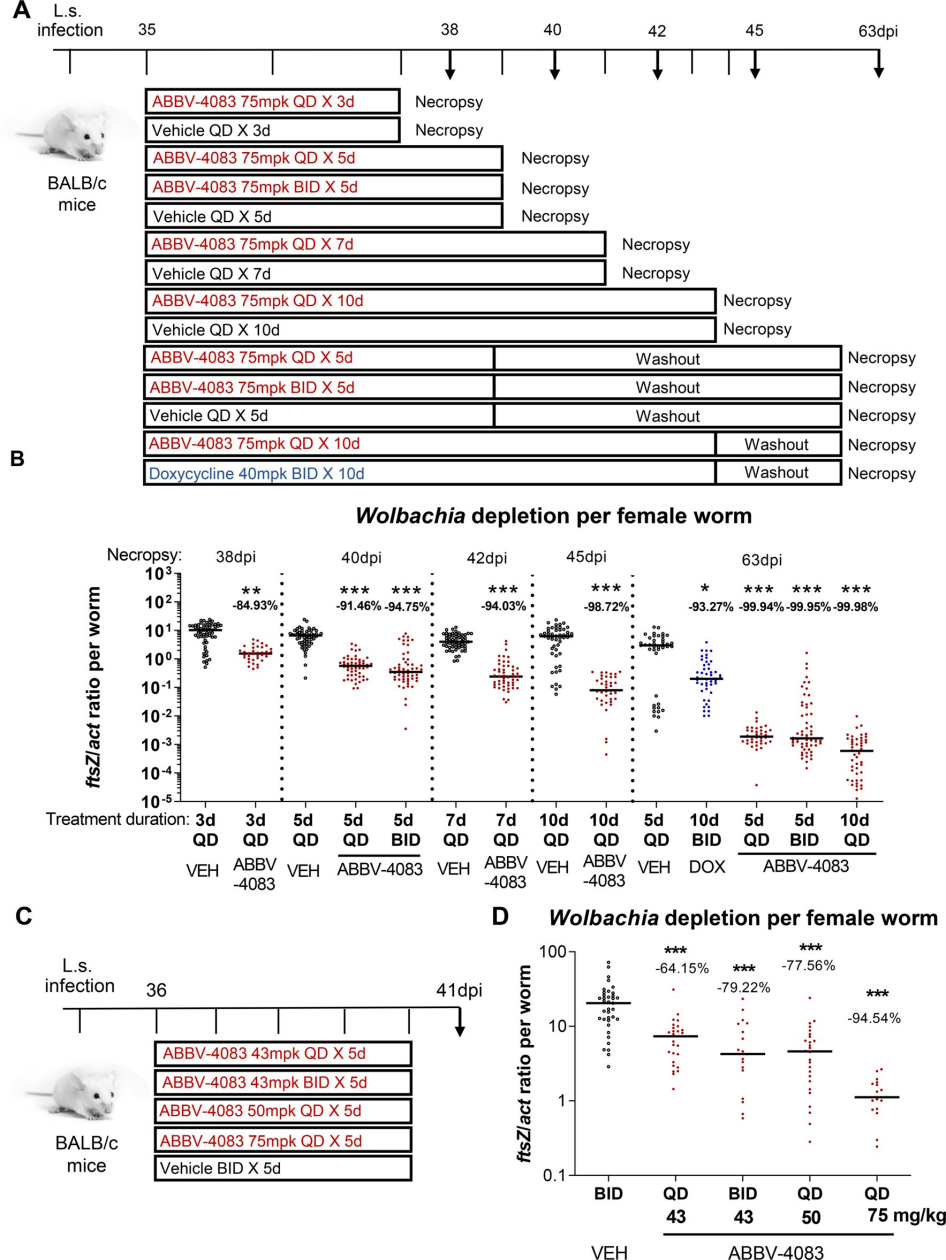
*Wolbachia* decline during and after ABBV-4083 treatment with twice daily treatment showing no significant superiority over once daily treatment. A, experimental design. 8-week-old female mice (n = 5–8 mice/group) were infected naturally with *L*. *sigmodontis*. At 35dpi the animals were treated orally either with ABBV-4083 (1x (QD) or 2x (BID) 75mg/kg (mpk) per day) for 3, 5, 7 or 10 days with necropsies at 38, 40, 42, 45 & 63dpi or doxycycline (DOX, 2x 40mg/kg per day) for 10 days and necropsy at 63dpi. Controls received an equal amount of vehicle (VEH, necropsy 38, 40, 42, 45 or 63dpi). B, *Wolbachia ftsZ*/*act* per female adult worm was quantified by real-time PCR (38dpi: VEH 3d = 60 worms; ABBV-4083 QD 3d = 36 worms; 40dpi: VEH 3d = 57 worms; ABBV-4083 QD 5d = 52 worms; ABBV-4083 BID 5d = 59 worms; 42dpi: VEH 7d = 59 worms; ABBV-4083 QD 7d = 52 worms; 45dpi: VEH 10d = 51 worms; ABBV-4083 QD 10d = 41 worms; 63dpi: VEH 5d = 43 worms; ABBV-4083 QD 5d = 37 worms; ABBV-4083 BID 5d = 60 worms; ABBV-4083 QD 10d = 47 worms, DOX BID 10d = 48 worms). C, experimental design. 8- to 10-week-old female mice (n = 5 mice/group) were infected naturally with *L*. *sigmodontis* and treated orally with ABBV-4083 (1x 43mg/kg; 2x 43mg/kg; 1x 50mg/kg; 1x 75mg/kg per day) or VEH for 5 days starting 36dpi with necropsies at 41dpi. D, *Wolbachia ftsZ*/*act* per female worm (VEH 5d = 37 worms; ABBV-4083 43mg/kg QD 5d = 27 worms; ABBV-4083 43mg/kg BID 5d = 19 worms; ABBV-4083 50mg/kg QD 5d = 26 worms; ABBV-4083 75mg/kg QD 5d = 17 worms). Data were tested for normal distribution by d‘Agostino & Pearson test. Statistical significance of not normally distributed data (B, D) was analyzed by Kruskal-Wallis followed by Dunn’s multiple comparison test. The horizontal lines indicate the median. p*<0.05, **p<0.01, ***p<0.001, compared with VEH.

To assess the kinetics of *Wolbachia* depletion in *L*. *sigmodontis* adult filariae during and subsequent to ABBV-4083 treatment, we conducted three studies in *L*. *sigmodontis*-infected mice. In the first, animals were treated at 35dpi with either a high dose (75mg/kg QD) of ABBV-4083 previously shown to be effective or vehicle QD for 3 or 7 days (Fig 1A). Necropsies were performed in the ABBV-4083 or vehicle treated animals one day after the last treatment (38, 42dpi, respectively). ABBV-4083 concentrations in blood obtained at two separate time points after oral dosing in mice were comparable to those obtained in satellite pharmacokinetic studies at the same dose [18]. In the second study, two additional dosing durations were examined to more completely assess the acute kinetics of *Wolbachia* depletion. In addition, the impact of a washout period after the end of treatment was assessed. Mice received 75mg/kg ABBV-4083 for 5 days QD (1x 75mg/kg per day) with necropsies either one day after the last treatment (40dpi) or a washout period of ~3 weeks (63dpi; Fig 1A). Additional groups received ABBV-4083 QD for 10 days and were analyzed at 45dpi (no washout) and 63dpi (washout). Separate vehicle controls for each necropsy day and doxycycline controls (40mg/kg BID 10 days) with necropsy at 63dpi were included.

ABBV-4083 treatment reduced endosymbiotic *Wolbachia* in a treatment duration dependent manner that correlated across both studies. After treatment end, 3, 5, 7 or 10 days of QD treatment reduced the *Wolbachia ftsZ*/*act* ratio compared to the respective vehicle control by 84.9%, 91.5%, 94.0% and 98.7%, respectively (Fig 1B). *Wolbachia* levels continued to decline during the washout period, and at 63dpi, the 5- and 10-day QD treatment regimens reduced the *Wolbachia ftsZ*/*act* ratio compared to the respective vehicle control by 99.94% and 99.98%, respectively. All differences in *Wolbachia* burden by ABBV-4083 treatment compared to the respective vehicle controls were statistically significant (p<0.01 after 3d treatment; p<0.001 after 5, 7, 10d treatment). Taken together, these results indicate that the *Wolbachia* levels decline in an approximately log-linear fashion between 3 and 10 days of treatment with ABBV-4083 and continue during the weeks following the end of treatment. As observed previously [18], control animals receiving a suboptimal 10-day doxycycline treatment exhibited a significantly smaller reduction in *Wolbachia ftsZ*/*act* ratio (93.3%, p<0.001) compared to animals treated with ABBV-4083 for the same duration (10 days).

### Effects of QD vs. BID dosing of ABBV-4083 on *Wolbachia* decline in *L*. *sigmodontis* infected mice

Two additional arms in the second study above allowed the comparison of 5 days of BID dosing of ABBV-4083 to either 5 or 10 days of QD dosing, with necropsies either one day after the last treatment or after a washout period (Fig 1A). At treatment end, 75mg/kg ABBV-4083 twice daily for 5 days produced a decline in *Wolbachia* only marginally better (94.8% compared to the respective vehicle control) than the same dose once daily for 5 days (91.5%, p>0.99), and inferior to the same total dose given over 10 days once daily (98.7%, p<0.05, Fig 1B). Because of the high (>99.9%) responses at this dose, no statistically significant differences between ABBV-4083 dosing regimens were observed after the washout period.

A third study in mice also compared QD and BID regimens, but at lower doses. Mice received 5 days of ABBV-4083 treatment with one of four regimens (43mg/kg QD, 43mg/kg BID, 50mg/kg QD or 75mg/kg QD) at 36dpi and were necropsied at day 41dpi (Fig 1C). Vehicle controls received 5 days of BID treatments. Five days of ABBV-4083 treatment reduced the *ftsZ*/*act* ratio in female worms in a dose dependent manner analyzed one day after treatment end at 41dpi (Fig 1D). ABBV-4083 treatment at 43mg/kg QD reduced the *Wolbachia ftsZ*/*act* ratio in female adult worms by 64.2%, at 43mg/kg BID by 79.2%, at 50mg/kg QD at 77.6% and at 75mg/kg QD at 94.5% compared to vehicle treated controls. All differences in *Wolbachia* burden were statistically significant in comparison to vehicle controls (p<0.001).

The above results indicate that in *L*. *sigmodontis*-infected mice, five days of once daily dosing of ABBV-4083 at 75mg/kg are sufficient to reduce *Wolbachia* levels by >90% and that depletion continues to >99% after a washout period. At both this and a lower dose, BID treatment led to a small, non-significant improvement in *Wolbachia* depletion in comparison to QD treatment regimens for the same duration.

### *Wolbachia* depletion in microfilariae coincides with the depletion in female adult worms

**Fig 2:**
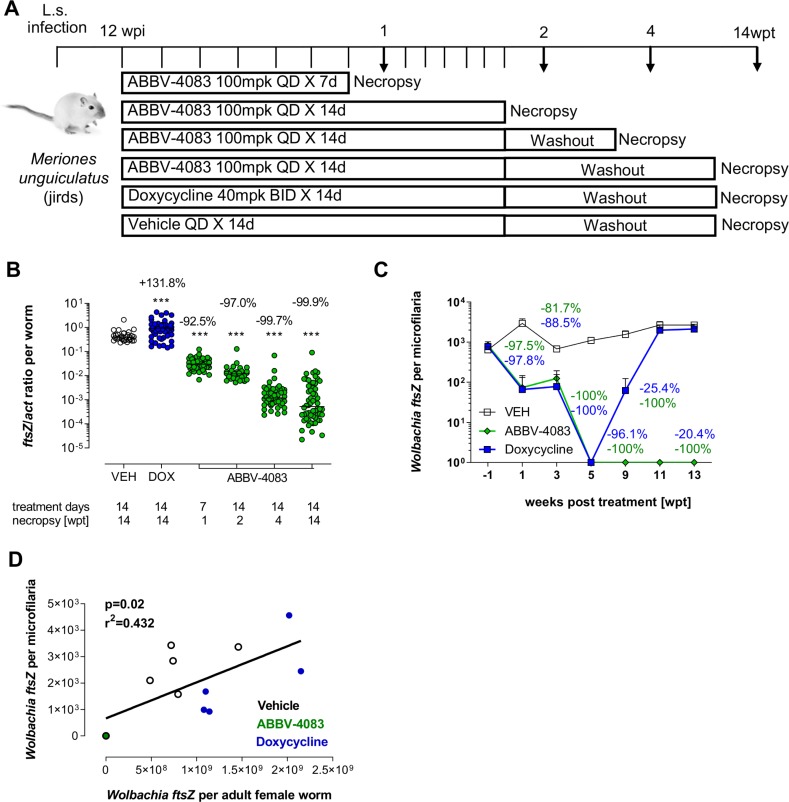
*Wolbachia* depletion in microfilariae coincides with the depletion in female adult worms. A, experimental design. Female jirds were infected naturally with *L*. *sigmodontis* and at 12wpi treated orally with ABBV-4083 (1x (QD) 100mg/kg (mpk) per day, 7 or 14 days, n = 6) or doxycycline (DOX, 2x (BID) 40mg/kg per day, 14 days, n = 6). Vehicle treated animals served as controls (VEH, n = 6). B, *Wolbachia ftsZ*/*act* ratio of isolated female adult worms after necropsy at 1, 2, 4, and 14 weeks after treatment (VEH = 30 worms; DOX = 53 worms; ABBV-4083 1wpt = 54 worms; ABBV-4083 2wpt = 30 worms; ABBV-4083 4wpt = 51 worms; ABBV-4083 14wpt = 58 worms). Data were tested for normal distribution by d‘Agostino & Pearson test. Statistical significance of not normally distributed data in B was analyzed by Kruskal-Wallis followed by Dunn’s multiple comparison test. The line indicates the median. ***p<0.001, compared with VEH. C, *Wolbachia ftsZ*/microfilaria over time (Mean + SEM) and percentage reduction compared with VEH for doxycycline (blue) and ABBV-4083 (green) 14-day treated groups. D, Spearman correlation of *Wolbachia ftsZ*/microfilariae and median *ftsZ*/female adult worms from the same animals at 14wpt.

While the adult worm burden starts to naturally decline in *L*. *sigmodontis* infected BALB/c mice ~70dpi, ~3 weeks after the development of microfilaremia [20], jirds are more permissive to long-term infections with *L*. *sigmodontis*, allowing an analysis for >5 months during patent (microfilariae-positive) infections [20]. We therefore assessed the effects of ABBV-4083 treatment on the *Wolbachia* depletion kinetics in female adult worms in jirds and compared it to the depletion observed in microfilariae, to ascertain the potential of *Wolbachia* depletion in microfilariae as a clinical surrogate indicator. Microfilariae positive jirds were treated QD with 100mg/kg ABBV-4083 for 7 or 14 days with necropsies at 1, 2, 4 and 14wpt. Controls received an equal volume of vehicle for 14 days QD or a suboptimal treatment with doxycycline (40mg/kg BID, 14 days) and were analyzed at 14wpt (Fig 2A).

Treatment with ABBV-4083 reduced the *Wolbachia* levels in female adult worms in a time-dependent manner (Fig 2B). At 1, 2 and 4wpt, 92.5%, 97.0% and 99.7% of *Wolbachia* were depleted, respectively, in comparison to the vehicle controls. After an extended washout (14wpt) the *Wolbachia* levels remained 99.9% depleted. Suboptimal two weeks of doxycycline treatment were not sufficient for long-term clearance of the *Wolbachia*: a 132% increase was observed in this group compared to the vehicle controls at 14wpt.

In accordance with the *Wolbachia* depletion in female adult worms, *Wolbachia ftsZ* levels in microfilariae of the ABBV-4083 and doxycycline treated groups were reduced by 1wpt by 97.5 and 97.8%, respectively, with only one out of six animals in both groups having detectable *Wolbachia* in microfilariae (Fig 2C). At 5wpt microfilariae from all 14-day ABBV-4083 and 14-day doxycycline treated animals had no detectable *Wolbachia ftsZ*. Whereas ABBV-4083 treated animals continued to have no detectable *Wolbachia ftsZ* levels until the end of the analysis at 13wpt, 1 out of 6 doxycycline treated animals had detectable *Wolbachia ftsZ* levels in microfilariae at 9wpt again and by 11 and 13wpt all microfilariae of doxycycline treated animals had detectable *Wolbachia ftsZ* levels (mean *Wolbachia ftsZ* reduction of 96.1%, 25.4% and 20.4% at 9, 11 and 13wpt), reaching levels equivalent to the start of the study.

These data indicate that *Wolbachia* depletion in microfilariae temporally correlates with the depletion in female adult worms (p = 0.02; r^2^ = 0.432 at 13/14wpt; Fig 2D), presenting a potential surrogate marker for the efficacy of *Wolbachia*-targeting compounds.

### Kinetics of ABBV-4083-induced clearance of microfilaremia and inhibition of filarial embryogenesis

In addition to the depletion kinetics of *Wolbachia* in adult worms and microfilariae, the impact of ABBV-4083 treatment on the clearance of peripheral microfilariae along with the inhibition of the embryogenesis of female adult worms over time was assessed.

**Fig 3:**
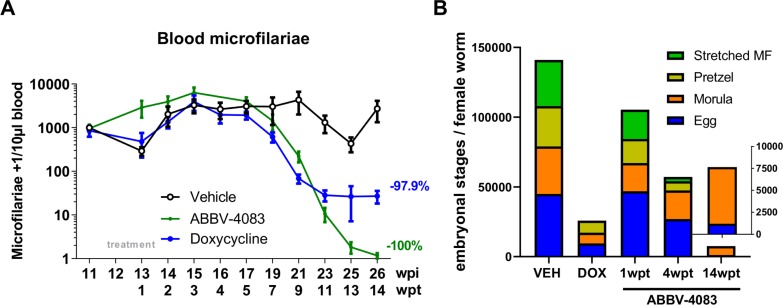
ABBV-4083 treatment clears microfilaremia and inhibits filarial embryogenesis. A, Microfilariae counts over time in *L*. *sigmodontis* infected jirds that were treated for 14 days with ABBV-4083 (1x 100mg/kg per day, n = 6), doxycycline (DOX, 2x 40mg/kg per day, 14 days, n = 6) or (1x VEH per day, n = 6) starting at 12 wpi. Data are presented as mean + SEM. B, embryograms from female adult worms isolated from *L*. *sigmodontis* infected jirds treated orally either with ABBV-4083 (1x 100mg/kg per day, 7 or 14 days, n = 6 jirds), doxycycline (DOX, 2x 40mg/kg per day, 14 days, n = 6 jirds) or vehicle (VEH, n = 6 jirds) at the time of necropsy 1, 4, 14 wpt (VEH = 10 worms; DOX = 20 worms; ABBV-4083 1wpt = 25 worms; ABBV-4083 4wpt = 20 worms; ABBV-4083 14wpt = 25 worms). The following embryonic stages were counted: egg, morula, pretzel and stretched microfilaria (MF). Data are presented as median.

Before treatment start all jirds included in this study were microfilariae positive. At 11wpt the first animal treated for 14 days with ABBV-4083 (QD 100mg/kg) cleared all microfilariae from the peripheral blood. By 14wpt 5 out of 6 ABBV-4083 treated jirds were amicrofilaremic and the one remaining microfilariae positive jird had declined from 809 at baseline to 1 microfilaria / 10μl blood (Fig 3A). Consistent with previous findings [18], all suboptimal doxycycline treated animals were microfilariae positive at 14wpt.

In accordance with the peripheral blood microfilariae counts over time, no marked reduction of the embryogenesis was observed one week after treatment onset with ABBV-4083 (QD 100mg/kg), with similar ratios of eggs, morulae, pretzel and stretched microfilariae stages in the uteri of the analyzed worms as in the vehicle control (Fig 3B). Four weeks after treatment onset with ABBV-4083, a significant reduction in eggs (p<0.01), pretzel (p<0.05) and stretched microfilariae (p<0.01) stages occurred in comparison to the vehicle controls and at 14wpt, a complete lack of stretched microfilariae stages in the ABBV-4083 treated group was observed along with statistically significantly reduced early embryonal stages (eggs & pretzel: p<0.001, morulae p<0.01). In comparison, suboptimal 2 weeks of doxycycline treatment reduced the number of early embryonal stages (egg p<0.001 and morulae p<0.05) by 14wpt, whereas two female worms harbored stretched microfilariae at that time point. In summary, ABBV-4083 treatment in jirds cleared microfilariae from the peripheral blood, completely inhibited embryogenesis and reduced all embryonal stages by 14wpt.

### Sequential daily treatment with ABBV-4083 is not essential for depletion of *Wolbachia* and microfilariae in the *L*. *sigmodontis* rodent model

**Fig 4:**
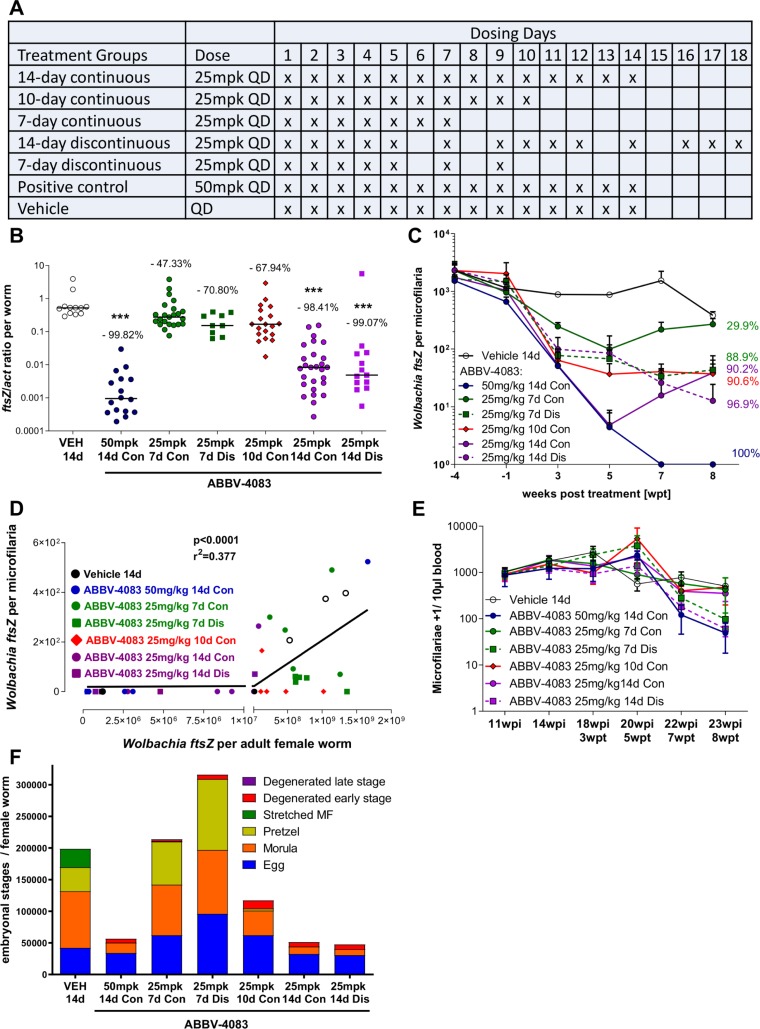
Continuous daily treatments with ABBV-4083 are not required for depletion of *Wolbachia* and microfilariae. A, experimental design. *L*. *sigmodontis*-infected jirds were treated orally either with ABBV-4083 1x 25mg/kg (mpk) QD consecutively for 7, 10 or 14 days (n = 6–7, consecutively (Con) treated group), or discontinuously (Dis) for 7 days total with skipped treatments on days 6 and 8 or discontinuously for 14 days with skipped treatments on days 6, 8, 13, and 15 (n = 6). Vehicle treated animals served as negative controls (VEH, n = 7), ABBV-4083 1x 50mg/kg for 14 day treated animals served as positive control. B, *Wolbachia ftsZ*/*act* ratio of isolated female adult worms at 8 weeks after treatment start (VEH = 12 worms; ABBV-4083 50mg/kg Con = 16 worms; ABBV-4083 25mg/kg 7d Con = 23 worms; ABBV-4083 25mg/kg 7d Dis = 9 worms; ABBV-4083 25mg/kg 10d Con = 19 worms; ABBV-4083 25mg/kg 14d Con = 27 worms; ABBV-4083 25mg/kg 14d Dis = 13 worms). Data were tested for normality by d‘Agostino & Pearson test. Statistical significance of not normally distributed data shown in B was analyzed by Kruskal-Wallis followed by Dunn’s multiple comparison test. ***p<0.001. C, *Wolbachia ftsZ*/microfilaria over time (mean + SEM). D, Spearman correlation of *Wolbachia ftsZ*/microfilariae and median *ftsZ*/female adult worms from the same animals at 8wpt. E, Microfilariae counts over time (mean + SEM) and F, embryograms from female adult worms (VEH = 6 worms; ABBV-4083 50mg/kg Con = 6 worms; ABBV-4083 25mg/kg 7d Con = 10 worms; ABBV-4083 25mg/kg 7d Dis = 1 worm; ABBV-4083 25mg/kg 10d Con = 7 worms; ABBV-4083 25mg/kg 14d Con = 16 worms; ABBV-4083 25mg/kg 14d Dis = 8 worms). The following embryonic stages were counted: egg, morula, pretzel and stretched MF, as well as degenerated early (egg, morula) and late (pretzel, stretched MF) stages. Data are presented as median.

Since strict adherence to multi-day drug administration can present a challenge to successful therapy, we assessed the impact of missed treatments on the efficacy of ABBV-4083 in a preclinical model. In this study three groups of jirds received 25mg/kg ABBV-4083 QD for 7, 10 or 14 consecutive days, respectively. Two other groups received discontinuous 7 and 14 treatments, skipping on days 6 and 8, and days 6, 8, 13 and 15, respectively, with once daily administration subsequently continuing to complete the regimen (Fig 4A). A lower, suboptimal dose of 25mg/kg ABBV-4083 was used in order to allow the identification of less prominent changes in the efficacy of continuous and discontinuous treatments. Positive controls received ABBV-4083 QD at 50mg/kg for 14 consecutive days and vehicle treated animals served as negative control.

Eight weeks after treatment start, jirds treated with ABBV-4083 QD at 50mg/kg for 14 days had a *Wolbachia* reduction of 99.8% in comparison to vehicle controls (Fig 4B). Lower doses of ABBV-4083 QD at 25mg/kg depleted the *Wolbachia ftsZ*/*act* ratio in a treatment duration dependent manner by 47.3%, 67.9% and 98.4% after 7, 10 and 14 consecutive days of treatment, respectively. Discontinuous daily treatment with 7 or 14 doses did not impair the *Wolbachia* depletions compared to continuous treatment, resulting in a reduction by 70.8% and 99.1%, respectively (Fig 4B). *Wolbachia* depletion in microfilariae at 8wpt (Fig 4C) correlated with the *Wolbachia* depletion in the adult worms (p<0.0001; r^2^ = 0.377, Fig 4D), reaching a mean *Wolbachia* reduction in microfilariae of jirds treated with ABBV-4083 QD at 50mg/kg for 14 days of 100% and at QD doses of 25mg/kg for 7, 10 and 14 consecutive days of 29.9% (6/6 animals with detectable *Wolbachia* in microfilariae), 90.6% (2/6 animals with detectable *Wolbachia* in microfilariae) and 90.2% (1/7 animals with detectable *Wolbachia* in microfilariae), respectively. Non-consecutive treatments for 7 and 14 days resulted in a *Wolbachia* reduction in microfilariae of 88.9% (4/5 animals with detectable *Wolbachia* in microfilariae) and 96.9% (1/6 animals with detectable *Wolbachia* in microfilariae), respectively.

Although this study was not designed to continue long enough to fully ascertain the effect on peripheral microfilaremia, microfilariae levels started to decline at 8 weeks after treatment start (Fig 4E), reaching statistical significance in animals treated for 14 days with 50mg/kg ABBV-4083 (p<0.01) and 25mg/kg ABBV-4083 on 14 non-consecutive days (p<0.05). Consistent with this trend and with the *Wolbachia* reduction shown in Fig 4B, later embryonal stages (pretzel and stretched microfilariae) were absent in the majority of female adult worms isolated from animals that received 14 days of treatment (Fig 4F). Reductions in later embryonal stages were independent of continuous or discontinuous ABBV-4083 treatment (Fig 4F; absence of pretzel stages: 14-day continuous group 68.7% (11/16 worms), 14-day discontinuous group 100% (8/8 worms), 50mg/kg group 100% (6/6 worms); absence of stretched microfilariae stages: 14-day continuous group absent in 93.7% (15/16 worms), 14-day discontinuous group 87.5% (7/8 worms), 50mg/kg group 100% (6/6 worms)). The population of the early embryonic morula stage was also reduced in the groups treated for 14 days (median reduction of 82% in 50mg/kg group; 87% in continuous group, 89% in discontinuous group) compared to vehicle or 7-day treated animals (median reduction in comparison to vehicle controls of 11% in continuous group and -13% in discontinuous group).

## Discussion

The present studies addressed several important aspects of the *in vivo* efficacy of the novel anti-*Wolbachia* agent ABBV-4083 [17, 18]. Using highly suppressive doses, the kinetics of *Wolbachia* depletion in female adult worms were investigated and the potential of using *Wolbachia* depletion in microfilariae as surrogate marker was ascertained. Using lower doses, the pharmacological aspects of more or less frequent dosing were analyzed, including comparisons of BID and QD treatment as well as the effect of nonsequential daily dosing as a model of incomplete adherence. These results provide useful guidance for the design of future clinical efficacy studies with ABBV-4083 and subsequent anti-*Wolbachia* candidates for the treatment of filariasis. One limitation of these studies is the use of an animal model employing a surrogate filarial nematode to model human filarial infections. Factors like the increased life expectancy of the human filarial nematodes, differences in *Wolbachia* densities and differences in the pharmacokinetics of ABBV-4083 in rodents and humans, as well as accessibility of the drug to the filariae (adult *L*. *sigmodontis* worms in the thoracic cavity vs. adult *O*. *volvulus* worms in subcutaneous nodules) may impact the translation of our results to human filarial infections. Furthermore, treatments in mice were performed before the occurrence of nematode patency, which will not be the case for clinical trials in filariasis patients. Nevertheless, our studies provide important information for designing aspects of a clinical program for ABBV-4083, including the rational selection of dosing regimens and the timing for investigation of *Wolbachia* depletion in microfilariae as a surrogate marker to ascertain both drug activity and possible bacterial recrudescence. Furthermore, although the filarial nematode utilized in our studies differs from the human pathogens, the target of drug activity (*Wolbachia*) is highly similar, giving relevance to the pharmacological aspects of ABBV-4083 investigated here. In addition, 200mg doxycycline for 4 weeks were previously shown to have significant macrofilaricidal efficacy in both lymphatic filariasis as well as onchocerciasis [1, 31, 32], indicating that drug exposures may be sufficient in the two anatomical sites (serous cavities/lymph and subcutaneous/deep tissue nodules) to mediate *Wolbachia* depletion. Future clinical studies will confirm the extent to which our findings are recapitulated in human clinical studies of ABBV-4083, and potentially with other anti-*Wolbachia* candidates.

One goal of the current studies was to characterize the kinetics of *Wolbachia* reduction in adult female worms after oral ABBV-4083 treatment. In both mice and jirds, the *Wolbachia* load declined in a time-dependent manner, beginning as soon as 3 days after treatment start and continuing through 14 days of treatment in an approximately log-linear fashion. *Wolbachia* levels continued to decline in the weeks following the discontinuation of treatment, a finding that was also observed for *in vitro Wolbachia* depletion in insect cells by doxycycline [33]. The depletion of *Wolbachia* was associated with profoundly disrupted embryogenesis, resulting in sterilization of adult female worms and the ablation of microfilariae production and release. The observed clearance of peripheral microfilaremia by ABBV-4083 was comparable to previous studies in *L*. *sigmodontis*-infected jirds using the anti-*Wolbachia* candidates ABBV-4083 and AWZ1066S starting at 8wpt and reaching complete absence of peripheral microfilariae by 12-15wpt [17, 18, 23]. Kinetic analysis of the embryogenesis indicated that as early as 4wpt the number of stretched microfilariae within the uteri were reduced by >90%, consistent with a half-life of the microfilariae within the peripheral blood of around 3–4 weeks [34] and a drop of peripheral microfilariae loads beginning around 8wpt.

In contrast to the complete absence of microfilariae after two weeks of ABBV-4083 treatment, suboptimal treatment for two weeks with BID doxycycline maximally lowered microfilariae levels by 98.6% at week 13, followed by the onset of a rebound by 14wpt, consistent with the recrudescence of *Wolbachia* observed in the adult worms from animals in that group. Similar results were observed in previous *L*. *sigmodontis* jird studies that used the suboptimal two-week regimen of doxycycline as a treatment time-matched control for ABBV-4083 and AWZ1066S [18, 23]. Similarly, human clinical studies have shown that at least 4 weeks of doxycycline therapy is required to achieve a macrofilaricidal effect in lymphatic filariasis and onchocerciasis patients [1, 8, 15, 31, 32]. These data indicate the importance of evaluating *Wolbachia* depletion over a period of several months in the jird model, as shorter observation periods may miss the recrudescence of *Wolbachia* and the rebound of microfilariae. Through use of this extended analysis period, our results confirm that two weeks of ABBV-4083 QD 75mg/kg treatment are superior in regard to *Wolbachia* depletion, microfilariae clearance and disruption of embryogenesis in comparison to a suboptimal two weeks of doxycycline treatment [18]. Since both ABBV-4083 (a macrolide) and doxycycline (a tetracycline) are expected to be bacteriostatic on the basis of inhibition of prokaryotic protein synthesis, the differences in kinetics observed in our studies is likely due to the substantial difference in *in vitro* potency between the two agents [18] rather than a consequence of mechanistic distinctions. The results of the current studies verify that the efficacy of ABBV-4083 is both dose- and treatment duration-dependent and provide the basis for exploration of 7- to 14-day regimens in clinical studies.

Importantly, the kinetics of *Wolbachia* depletion in female adult worms was recapitulated by the *Wolbachia* depletion in microfilariae. Thus, already at 1wpt, *Wolbachia* were reduced in the majority of microfilariae of doxycycline or ABBV-4083 treated animals and no *Wolbachia* were detectable by 5wpt in both groups. This coincided with a *Wolbachia* depletion of 92.5% and 99.7% in female adult worms at 1 and 4wpt of ABBV-4083 treated jirds, respectively. Similarly, while microfilariae of ABBV-4083-treated jirds had no detectable *Wolbachia* through to the end of the study at 13wpt, microfilariae from suboptimal doxycycline treated animals displayed a rebound of the *Wolbachia* similar to that observed in the adult worms. The measurement of *Wolbachia* levels in microfilariae may thus be a suitable indicator for the *Wolbachia* depletion and recrudescence in adult worms, allowing for similar kinetic studies on the *Wolbachia* in microfilariae during human clinical trials without the need for repeated surgical removal of the nodules harboring adult worms from onchocerciasis patients. Our laboratories have previously sampled microfilariae from the circulation of bancroftian filariasis patients at 3–4 months after commencement of 2–4 week doxycycline regimens in order to determine anti-*Wolbachia* efficacy. These clinical data indicate that average anti-*Wolbachia* depletion levels in microfilariae in patient cohorts shortly following drug removal are indicative of long-term macrofilaricidal activity, with declines of >90% being predictive of eventual significant curative activity at 18 months [14, 35, 36]. Our present data corroborate that sampling microfilariae in the periphery will likely be an acceptable surrogate indicator of anti-*Wolbachia* efficacy within adult filarial worms following treatment of the clinical candidate ABBV-4083, and thus may be predictive of long-term efficacy. A first proof of concept that *Wolbachia* can be determined from microfilariae of *O*. *volvulus* patients as well was previously demonstrated by our laboratory [37]. However additional work is needed to allow the measurement from human skin samples that contain only a single or few microfilariae.

The results of our studies suggest that BID vs. QD dosing of ABBV-4083 produces only a modest increase in efficacy, equivalent to a small increase in QD dose, despite the short plasma half-life of ABBV-4083 in mice [18]. Instead, increasing the number of days of QD dosing appears to have a greater effect on efficacy than BID dosing for a shorter duration. This observation is consistent with the fact that *Wolbachia* is a slowly replicating organism (doubling time 14h in insect cells [38] and much longer in filarial worms [39], where they cannot continue to multiply in adult worms without overloading and damaging their host) and the observations that *Wolbachia* levels continue to decline after dosing is completed. Thus, these studies provide support for investigation of a QD rather than BID dosing regimen of ABBV-4083 in clinical studies of efficacy irrespective of its half-life in humans.

Finally, in the context of a regimen of 7 or 14 daily doses of ABBV-4083, omitting 2 or 4 days of dosing, respectively, did not impair *Wolbachia* depletion in comparison to consecutive 7 or 14 daily treatments, provided that the missed daily doses were given after the originally planned end of treatment to complete the 7- or 14-day treatments with the regimen. This result may also be related to the slow replication rate of *Wolbachia*. This study was designed to mimic a pattern of incomplete adherence to a 7- or 14-day regimen in humans. The equivalent anti-*Wolbachia* efficacy of matched continuous and discontinuous groups in Fig 4B suggests that clinical efficacy may not be compromised by some missed doses, as long as the missed doses are given at the end of treatment, consistent with our clinical trials on doxycycline where we also allowed missed treatments to be completed at the end of treatment [15, 40, 41]. In this regard, instructing patients to complete the entire regimen with once daily dosing even if days are missed may be useful.

In conclusion, our results demonstrate that treatment duration rather than BID vs. QD treatment primarily determines the efficacy of ABBV-4083. Furthermore, they indicate that some degree of variable, incomplete adherence to the dosing regimen may be acceptable without seriously impairing the *Wolbachia* depletion efficacy, provided that the regimen is subsequently completed. The *Wolbachia* depletion was shown to occur within days after start of anti-*Wolbachia* therapy, and levels continued to decline during the weeks following the end of treatment. The depletion of *Wolbachia* is associated with profoundly disrupted embryogenesis, sterilization of adult female worms, and the ablation of microfilarial production and release. Finally, the correlation of *Wolbachia* decline and recrudescence between adult worms and microfilariae in this model provides a basis for exploration of *Wolbachia* levels in skin microfilariae as a surrogate indicator for anti-*Wolbachia* activity in *O*. *volvulus* infection.
